# High-Pressure High-Temperature Nanodiamond-Modified ZnO Nanocomposites as Promising Photocatalysts: Synthesis and Characterization

**DOI:** 10.3390/ma19030609

**Published:** 2026-02-04

**Authors:** Julia Micova, Natalia Kosutova, Miroslav Cavojsky, Anna Artemenko, Zdenek Remes, Bruno Masenelli, Gilles Ledoux

**Affiliations:** 1 Institute of Chemistry Slovak Academy of Sciences, Dúbravská cesta 5807/9, 845 38 Bratislava, Slovakia; natalia.kosutova@savba.sk; 2 Institute of Materials and Machine Mechanics Slovak Academy of Sciences, Dúbravská cesta 9/6319, 845 13 Bratislava, Slovakia; miroslav.cavojsky@savba.sk; 3 Institute of Physics of the Czech Academy of Sciences, Na Slovance 1999/2, 182 00 Prague, Czech Republic; artemenko@fzu.cz; 4 INSA Lyon, Ecole Centrale de Lyon, CNRS, Universite Claude Bernard Lyon 1, CPE Lyon, INL, UMR5270, 69621 Villeurbanne, France; bruno.masenelli@insa-lyon.fr; 5 Universite Claude Bernard Lyon 1, CNRS, Institut Lumière Matière, UMR5306, F-69100 Villeurbanne, France; gilles.ledoux@univ-lyon1.fr

**Keywords:** high-pressure and high-temperature NDs, ZnO nanocomposites, defect engineering, photocatalysis, methylene blue degradation, cathodoluminescence, XPS, Raman spectroscopy

## Abstract

**Highlights:**

**What are the main findings?**

- HPHT nanodiamonds enhance the performance of ZnO-based photocatalysts.
- Optimal ND:ZnO ratios suppress defects and improve charge-carrier separation.
- ND–ZnO 10 (ND:ZnO = 0.1 w/w) achieves threefold-higher photocatalytic efficiency compared to pristine ZnO.

**What are the implications of the main findings?**

- HPHT ND–ZnO composites open new directions for defect engineering in photocatalysis.
- HPHT ND–ZnO systems provide promising photocatalysts for environmental remediation.
- ND–ZnO 10 shows stable reuse, confirming its potential for water-treatment applications.

**Abstract:**

Zinc oxide (ZnO) nanostructures suffer from fast electron–hole recombination, limiting their applicability in photocatalytic environmental remediation, and carbon additives such as detonation nanodiamonds (DNDs) are constrained by their high defect density. To address this, ZnO nanocomposites modified with high-pressure, high-temperature nanodiamonds (HPHT NDs) were synthesized to evaluate whether their intrinsically lower defect density—evidenced by a dominant diamond Raman peak at 1330 cm^−1^ and a low sp^2^ carbon fraction of 6.6% compared to oxidized DNDs with strong D/G bands (~1350/1580 cm^−1^) and ~25–35% sp^2^ carbon—can enhance charge separation and improve photocatalytic activity. Oxidized HPHT NDs bearing carbonyl, carboxyl, and hydroxyl groups enabled covalent attachment to ZnO, and the resulting ND–ZnO composites were characterized by SEM/EDX, ATR-FTIR, Raman spectroscopy, XPS, and cathodoluminescence (CL). EDX confirmed increasing carbon incorporation from 13.0 to 52.9 at.%, while XPS revealed a 0.5 eV shift in the Zn 2p_3_/_2_ peak and an increase in Zn–O–Zn lattice oxygen from 31.3% to 61.6% in ND–ZnO 10. CL showed enhanced near-band-edge emission and reduced Zn*i*-related luminescence (~3.0 eV). ND–ZnO 10 achieved a nearly threefold-higher degradation rate constant (0.0251 min^−1^) than pristine ZnO (0.0087 min^−1^) and retained 88% efficiency after five cycles, demonstrating strong potential for durable wastewater treatment.

## 1. Introduction

Environmental pollution caused by persistent organic dyes has intensified the need for efficient and sustainable remediation technologies. Semiconductor photocatalysis offers a promising approach for degrading organic pollutants under light irradiation, providing an environmentally friendly alternative to conventional treatment methods. Zinc oxide (ZnO) nanostructures have attracted considerable attention as photocatalysts due to their wide direct band gap (~3.3 eV), high exciton binding energy, chemical stability, and favorable optical and electrical properties [1,2]. Moreover, hydrothermal synthesis of ZnO is simple, cost-effective, and suitable for large-scale production [3]. Despite these advantages, the photocatalytic efficiency of ZnO is significantly limited by the rapid recombination of photogenerated electron–hole pairs [4,5], which reduces the formation of reactive oxygen species essential for dye degradation. To overcome this limitation, numerous strategies have been explored, including metal doping [4,5], noble-metal deposition [4,5], and coupling with other semiconductors [4,5]. In recent years, carbon-based materials have emerged as effective modifiers for ZnO due to their ability to enhance charge separation and electron transport [6,7,8,9]. ZnO/carbon composites have demonstrated improved photocatalytic degradation of dyes such as rhodamine B and methylene blue under UV irradiation [10]. Systems incorporating carbon nanotubes (CNTs) [11], graphene-based materials [12,13,14,15], fullerenes [16], or carbon frameworks derived from metal–organic frameworks (MOFs) [17,18] have shown that carbon phases can suppress recombination and improve photocatalytic activity. ZnO has also been combined with carbon quantum dots (CQDs), which act as efficient electron acceptors and up-conversion agents, leading to enhanced charge separation and improved photocatalytic degradation of organic pollutants [19,20,21]. However, these materials often suffer from structural heterogeneity, high defect density, or limited long-term stability, which may hinder reproducibility and controlled defect engineering.

Nanodiamonds represent another class of carbon nanomaterials explored for ZnO modification. Detonation nanodiamonds (DNDs) have been incorporated into ZnO to enhance photocatalytic activity [22,23,24], but their highly defective structure, high sp^2^ carbon content, and chemically unstable surface limit their applicability [25,26]. In contrast, high-pressure high-temperature nanodiamonds (HPHT NDs) possess stable crystalline diamond cores, significantly lower defect density, and tunable surface chemistry [25,26]. These characteristics make HPHT NDs promising candidates for controlled defect engineering in ZnO, potentially enabling more efficient charge separation and reduced non-radiative recombination. Despite these advantages, the role of HPHT NDs in ZnO-based photocatalysts remains largely unexplored, and their influence on structural defects, optical properties, and photocatalytic performance has not been systematically investigated. To the best of our knowledge, no peer-reviewed studies on ZnO composites prepared with HPHT nanodiamonds have been published so far. Only a recent conference contribution has briefly mentioned ZnO decorated on HPHT nanodiamond substrates [27], indicating that this research direction is still in its early stage. This scarcity of research highlights the novelty and relevance of the present work.

In this study, we synthesize ZnO nanocomposites modified with oxidized HPHT nanodiamonds and evaluate their structural, optical, and photocatalytic properties. By tailoring the surface functionalities of HPHT NDs and integrating them with ZnO, we aim to clarify how these low-defect carbon nanostructures influence charge carrier dynamics and photocatalytic degradation of methylene blue. The results provide new insights into ZnO/carbon hybrid systems and demonstrate the potential of HPHT nanodiamonds as controlled and stable platforms for defect engineering in photocatalysis.

## 2. Materials and Methods

### 2.1. Materials

Zinc nitrate hexahydrate (Zn(NO_3_)_2_·6H_2_O, ≥99%), hexamethylenetetramine (C_6_H_12_N_4_, 99%), sodium hydroxide (NaOH, ≥98%), nitric acid (HNO_3_, 70%), hydrochloric acid (HCl, 37%), and methylene blue (MB, C_16_H_18_ClN_3_S·xH_2_O) were purchased from Merck and used without further purification. High-pressure high-temperature (HPHT) nanodiamonds (type Ib, ~30 nm) were obtained from Microdiamant AG (Lengwil, Switzerland). Deionized water (resistivity 18.2 MΩ·cm) was used in all experiments.

### 2.2. Photocatalyst Preparation

#### 2.2.1. Oxidation of HPHT Nanodiamonds

HPHT nanodiamonds (Microdiamant AG, Switzerland) were oxidized using a procedure adapted from previously published thermal and chemical oxidation methods for HPHT and detonation nanodiamonds [28,29,30]. In the first step, the nanodiamonds were thermally oxidized in air at 500 °C for 6 h. Subsequently, chemical oxidation was performed in concentrated nitric acid (70% HNO_3_, Merck KGaA, Darmstadt, Germany) under sonication (80 kHz) to introduce carbonyl surface groups. The suspension was diluted with deionized water (1:1, *v*/*v*) and sonicated for an additional 1 h. Nanodiamonds were separated by centrifugation, washed sequentially with 0.1 M NaOH and 0.1 M HCl, rinsed with Milli-Q water until neutral pH, and dried by lyophilization.

#### 2.2.2. Preparation of ZnO Nanostructures

ZnO nanostructures were synthesized by a hydrothermal method as stated by [31]. Equimolar aqueous solutions of zinc nitrate hexahydrate (25 mM) and hexamethylenetetramine (25 mM) were mixed and heated at 90 °C for 3 h. The precipitate was collected, washed three times with deionized water, and centrifuged at 18,000 rpm for 15 min to remove residual salts. The obtained powder was dried in air at 130 °C for 20 h.

#### 2.2.3. Preparation of ND–ZnO Composites

ND–ZnO composites were prepared with ND:ZnO weight ratios of 0.05, 0.10, 0.15, and 0.20. Oxidized nanodiamonds were dispersed in 20 mL of deionized water and sonicated for 30 min. ZnO powder was then added, and the suspension was stirred for 5 h and left to react overnight at room temperature. The resulting powders were collected by centrifugation (12,500 rpm, 15 min), washed with deionized water, and dried in air at 130 °C for 20 h. The samples were designated ND–ZnO 5, ND–ZnO 10, ND–ZnO 15, and ND–ZnO 20.

**Figure 1:**
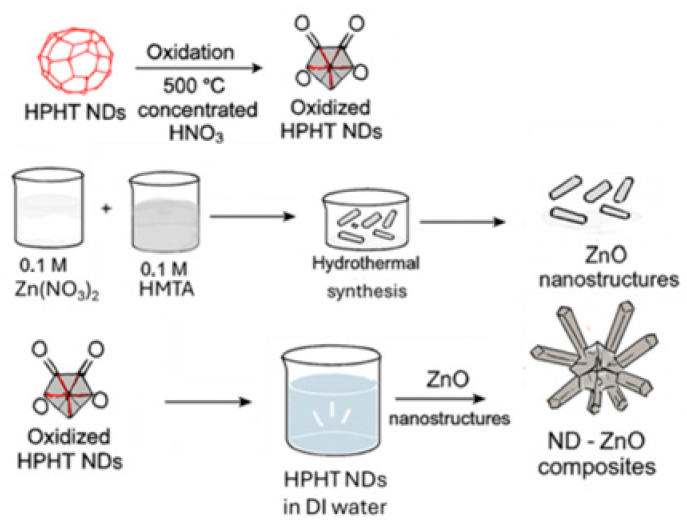
Overview of the synthetic pathway leading to ND-ZnO nanocomposites, which includes HPHT oxidation of NDs, hydrothermal formation of ZnO, and assembly of the composite.

The preparation procedure of ND-ZnO nanocomposites is schematically shown in Figure 1.

### 2.3. Structural Characterization

#### 2.3.1. SEM and EDX Analysis

The morphology and elemental composition of the ND–ZnO nanocomposites were examined using a scanning electron microscope (Phenom G2 Pro, Thermo Fisher Scientific, Waltham, MA, USA) equipped with an energy-dispersive X-ray (EDX) detector, operated at 10 kV. SEM images and EDX spectra were acquired and processed with the Phenom^TM^ Pro Suite software (product information available at: https://www.thermofisher.com/ (accessed on 22 January 2026)). Samples were prepared by depositing powders onto aluminum stubs with silver paint and dried at room temperature before imaging.

#### 2.3.2. Vibration Spectroscopy (FTIR and Raman)

Infrared spectra were recorded in the mid-IR region (500–4000 cm^−1^) using a Fourier transform infrared spectrometer (iS50, Thermo Fisher Scientific, USA) equipped with a diamond ATR prism. Raman spectra were obtained with a DXR Raman Microscope (Thermo Fisher Scientific, Waltham, MA, USA) using a 532 nm laser at 10 mW excitation power. Measurements were performed at room temperature, and peak positions were calibrated with a polystyrene standard.

#### 2.3.3. X-Ray Photoelectron Spectroscopy (XPS)

Surface chemical composition was determined by X-ray photoelectron spectroscopy (AXIS Supra, Kratos Analytical Ltd., Manchester, UK) with a monochromated Al Kα source (1486.6 eV). Survey spectra were collected at a pass energy of 80 eV and high-resolution spectra at 20 eV. Binding energies were calibrated to the C–C bonds of the C 1s peak at 285.0 eV according to works [28,32,33]. Peak fitting was performed using CasaXPS (version 2.3.16 PR 1.6) software with Gaussian/Lorentzian line shapes.

#### 2.3.4. XRD Characterization

X-ray diffraction (XRD) measurements on powder samples of as-grown ZnO, oxidized HPHT nanodiamonds, and ND–ZnO composites with varying ND loadings were performed using a Rigaku MiniFlex 600 diffractometer (Rigaku Corporation, Tokyo, Japan) equipped with Cu Kα_1_,_2_ radiation. The angular range was 10–90° with a step size of 0.02°.

#### 2.3.5. Cathodoluminescence (CL) Spectroscopy

Cathodoluminescence spectra were measured using an SEM (MIRA 3, TESCAN GROUP, Brno, Czech Republic) equipped with a MonoCL4 module (Gatan, Pleasanton, CA, USA). The acceleration voltage was set to 10 kV with a beam current of ~1 nA. Spectra were collected in the range of 300–800 nm and corrected for system response.

### 2.4. Photocatalytic Activity Experiments

The photocatalytic activity of the ND–ZnO composites was evaluated by the degradation of methylene blue (MB) under UV irradiation. In a typical experiment, 20 mg of photocatalyst was dispersed in 15 mL of MB aqueous solution (10 mg/L) and stirred in the dark for 30 min to establish the adsorption–desorption equilibrium. The suspension was then irradiated with a UV LED source (M365L3, Thorlabs, Newton, NJ, USA; λ = 365 nm, power density 150 mW·cm^−2^). At regular 30 intervals, 2 mL aliquots were withdrawn and filtered through a PVDF membrane (pore size 0.22 μm). The residual MB concentration was determined by measuring the absorbance at 662 nm using a UV-Vis spectrophotometer (DU 730, Beckman Coulter, Brea, CA, USA). For comparison, the photocatalytic performance of pristine ZnO nanostructures was evaluated under identical conditions.

#### Reusability Test

The reusability of the most efficient composite (ND–ZnO 10) was evaluated to confirm its photocatalytic stability during the degradation of methylene blue (MB). In the first cycle, 20 mg of the composite was dispersed in 15 mL of MB solution (10 mg/L) and stirred in the dark for 30 min to establish adsorption–desorption equilibrium. The suspension was then irradiated with UV light for 120 min under the same conditions as described in Section 2.4. After each cycle, the catalyst was recovered by centrifugation at 12,500 rpm for 15 min, washed with deionized water, and dried in air at 130 °C for 2 h before reuse. The recovered ND–ZnO 10 composite was reused in five consecutive cycles under identical experimental conditions.

## 3. Results

### 3.1. Structural Analysis (SEM and EDX)

**Figure 2:**
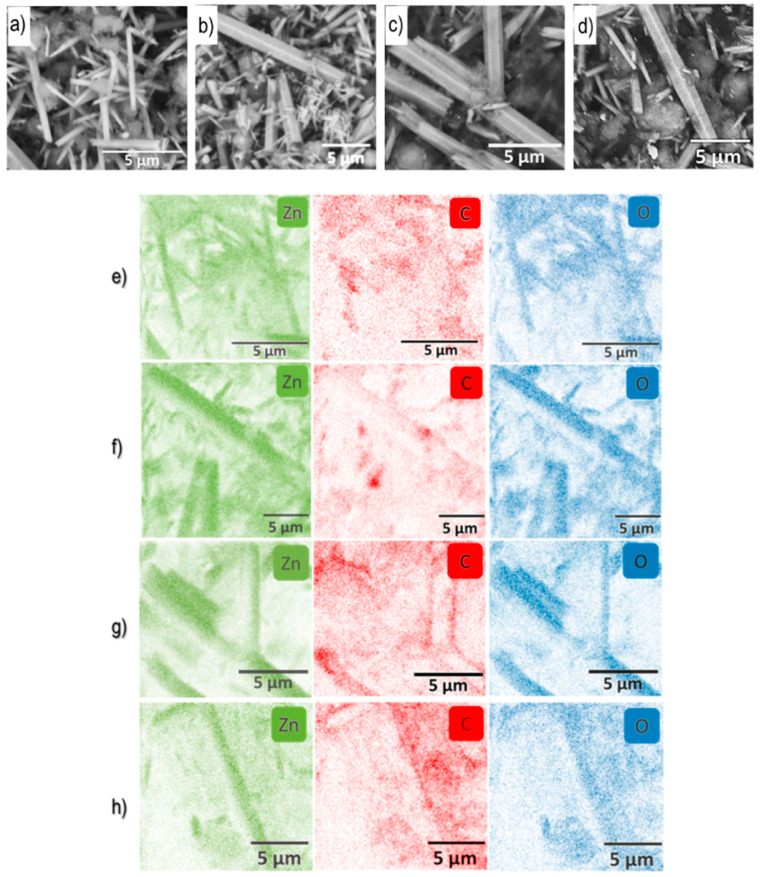

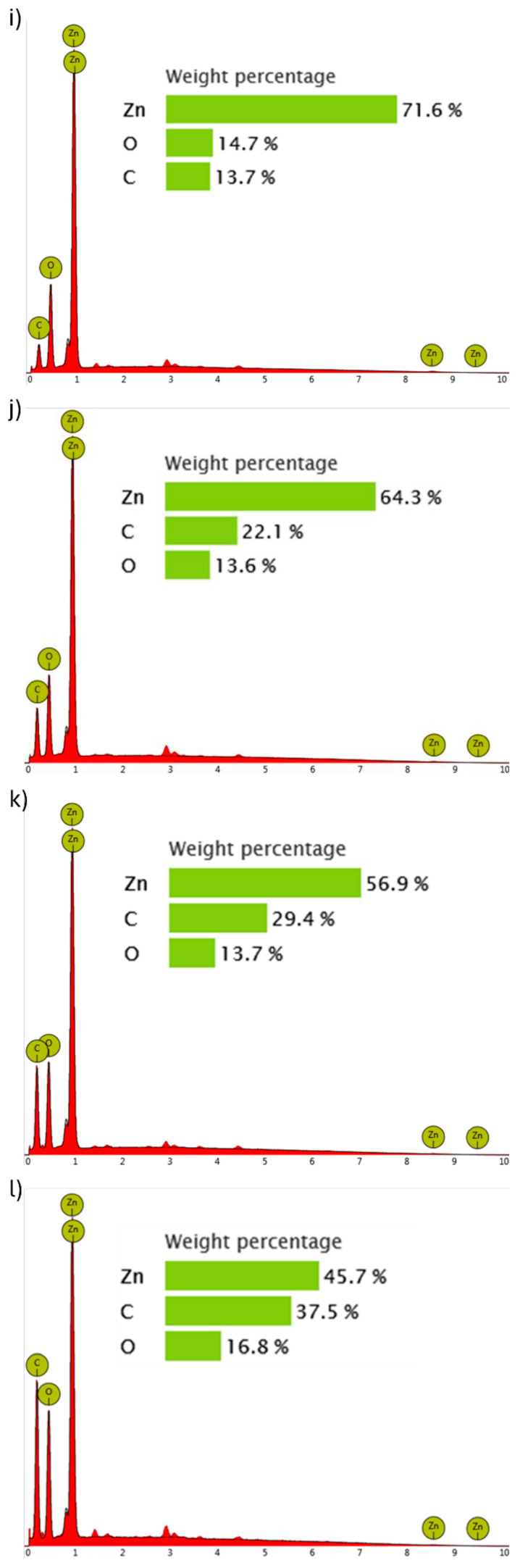
Scanning electron microscopy (SEM) images of the ND-ZnO composites: (**a**) ND-ZnO 5, (**b**) ND-ZnO 10, (**c**) ND-ZnO 15, (**d**) ND-ZnO 20. EDX elemental mapping of the ND-ZnO composites: (**e**) ND-Zn5 10, (**f**) ND-ZnO 10, (**g**) ND-ZnO 15, (**h**) ND-ZnO 20. Quantitative EDX analysis of the ND-ZnO composites: (**i**) ND-ZnO 5, (**j**) ND-ZnO 10, (**k**) ND-ZnO 15, (**l**) ND-ZnO 20.

SEM images (Figure 2a–d) revealed that the ND–ZnO composites predominantly consisted of ZnO nanorods with varying degrees of surface roughness and aggregation depending on the ND content. Increasing ND loading led to more irregular morphologies, indicating increased surface disorder and possible defect formation.

EDX elemental mapping (Figure 2e–h) confirmed the homogeneous distribution of Zn, O, and C across all samples, with no visible phase segregation. The Zn signal (green) was dominant in the sample ND–ZnO 5, while the C signal (red) intensified progressively with higher ND content. Oxygen (blue) remained relatively stable across the series, indicating preservation of the ZnO lattice framework.

Quantitative EDX analysis (Figure 2i–l) showed a systematic decrease in Zn content from 71.6 wt.% in sample ND–ZnO 5 to 45.7 wt.% in the ND–ZnO 20 composite. Concurrently, the carbon content increased from 13.7 wt.% to 37.5 wt.%, reflecting successful incorporation of nanodiamond species. Oxygen levels fluctuated slightly (14.7–16.8 wt.%) but remained within expected ranges for ZnO-based systems.

### 3.2. Vibration Spectroscopy (Raman and FTIR)

**Figure 3:**
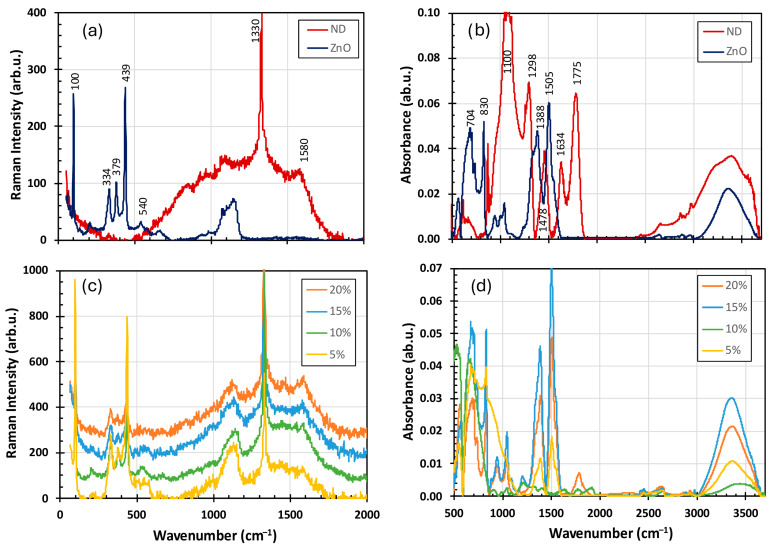
Raman (**a**,**c**) and optical absorbance (**b**,**d**) spectra of NDs, ZnO reference, and samples of composites ND-ZnO.

The Raman spectrum of the reference ND sample (Figure 3a) exhibited the characteristic diamond peak at 1330 cm^−1^ and the G band at ~1580 cm^−1^, which corresponded to the in-plane stretching vibrations of sp^2^-bonded carbon atoms in graphitic structures [34]. The G band, a signature of graphitic carbon, was present in both ordered and disordered carbon phases. In the reference ZnO sample, the Raman peak at 100 cm^−1^ was assigned to the nonpolar E2(Low) phonon mode of the Zn sublattice. In comparison, the peak at 439 cm^−1^ correspoinded to the nonpolar E2(High) phonon mode of the oxygen sublattice [35]. Additional peaks at 334 and 540 cm^−1^ were attributed to the sum and differential modes E2(High) ± E2(Low) [36], and the band at ~379 cm^−1^ was assigned to the A1 transverse optical (TO) phonon mode of the wurtzite ZnO structure [37]. A weak Raman band in the 1070–1170 cm^−1^ region was associated with multiphonon processes and combinations of acoustic modes with A1 and E2 symmetry.

The FTIR spectra (Figure 3b) revealed a broad absorption band in the 560–860 cm^−1^ region, corresponding to first- and second-order longitudinal optical modes of the ZnO lattice [38]. The band at 1300–1500 cm^−1^ was assigned to bending vibrations of surface hydroxyl groups [39]. In nanodiamonds, the absorption band at ~1100 cm^−1^ was attributed to C–O stretching vibrations, the peak at 1775 cm^−1^ to C=O stretching vibrations of carbonyl groups, the bands at 2850–2950 cm^−1^ to C–H stretching vibrations, and the broad band at 3200–3600 cm^−1^ to O–H stretching vibrations of hydroxyl groups [29].

The Raman spectra of the ND–ZnO composites (Figure 3c) displayed contributions from both ND and ZnO. In the ND–ZnO 5 sample, the G band at ~1580 cm^−1^ was suppressed. The FTIR spectra of the composites (Figure 3d) exhibited significant variations, with diamond-related absorption bands largely absent. A prominent feature across the composites was the broad O–H stretching band at 3200–3600 cm^−1^. In the ND–ZnO 10 sample, the reduction in the bands in the 1300–1500 cm^−1^ region indicated a lower contribution of hydroxyl groups.

### 3.3. X-Ray Photoelectron Spectroscopy (XPS)

XPS spectra confirmed the presence of Zn, O, and C in all composites. Increasing ND content led to higher carbon concentrations and lower zinc contributions (Figure 2i–l). XPS measurements revealed that the ZnO reference contained approximately 2.5 at.% of N (Figures S1 and S2) on its surface. A relatively high C concentration (~33 at.%) was detected on the surface of the ZnO reference sample, likely due to contamination from the air. Also, the processing of XPS data showed that sample oxidized NDs had low traces of Si (~ 0.6 at.%) on the surface.

In Table 1, Table 2, Table 3 and Table 4, the atomic concentrations (Table 1) are derived from high-resolution XPS spectra after Shirley background subtraction and sensitivity-factor correction. In Table 2, Table 3 and Table 4, the values reported as ‘concentration of chemical bonds (%)’ represent the relative peak-area contributions obtained from deconvolution of the Zn 2p, O 1s, and C 1s spectra. These percentages do not correspond to atomic concentrations but to the normalized fractional areas of individual fitted components. Peak assignments and binding-energy positions follow the literature references [28,30,32,40,41], and all spectra are calibrated to the C–C/C–H component of the C 1s peak at 285.0 eV.

**Figure 4:**
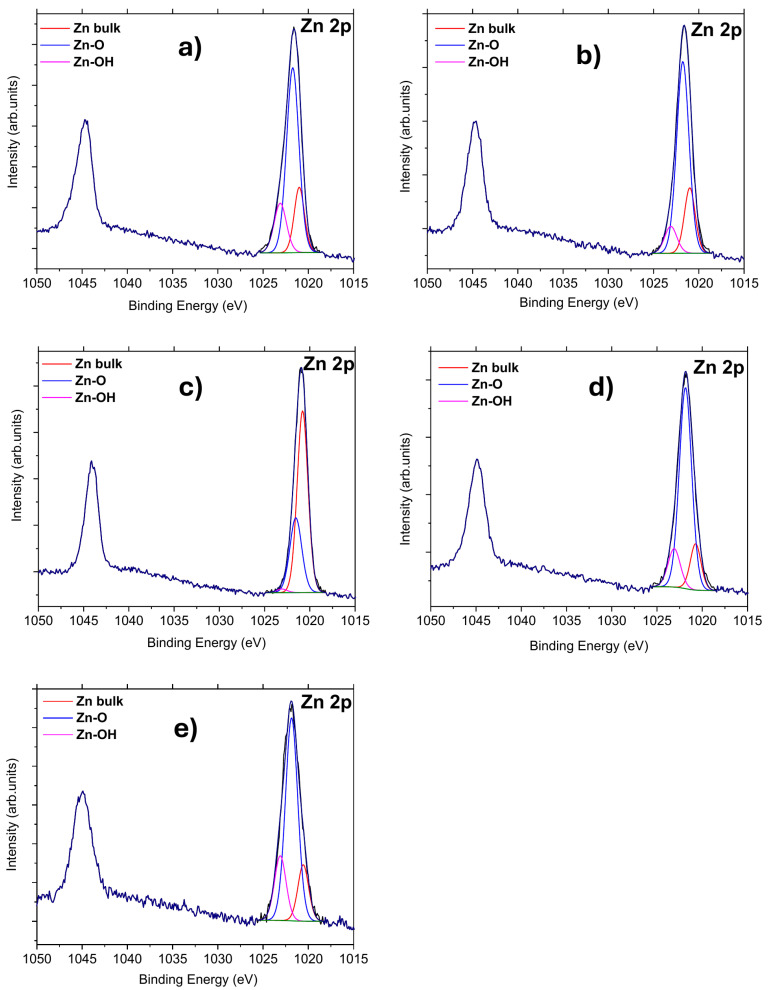
The Zn 2p XPS peaks of (**a**) reference ZnO, (**b**) ND-ZnO 5, (**c**) ND-ZnO 10, (**d**) ND-ZnO 15, and (**e**) ND-ZnO 20 samples. The green line is the background line of fitted spectra.

High-resolution Zn 2p spectra (Figure 4, Table 2) of the ZnO reference sample exhibited the characteristic spin–orbit doublet peaks Zn 2p_3_/_2_ (1021.6 eV) and Zn 2p_1_/_2_ (1044.6 eV), separated by 23.0 eV. The binding energies of the Zn 2p peaks corresponded to the Zn^2+^ oxidation state in ZnO [42]. The shape and position of the Zn 2p peaks in the ZnO reference and ND–ZnO 5, 15, and 20 samples were similar. In contrast, the Zn 2p_3_/_2_ peak of ND–ZnO 10 was shifted by 0.5 eV to a lower binding energy compared to the ZnO reference. The Zn 2p spectra were deconvoluted into three components [32]: 1020.8 eV (Zn bulk), 1021.6 ± 0.2 eV (Zn–O), and 1023.3 eV (Zn–OH).

As summarized in Table 2 and Figure 4, the ND–ZnO powder composites (except ND–ZnO 20) exhibited a reduction in Zn–OH contributions compared to the reference ZnO sample. Moreover, the concentration of bulk Zn bonds was higher in the mixed composites (except ND–ZnO 5) than in the ZnO reference. Notably, the concentration of Zn–O bonds in ND–ZnO 10 was almost two times lower than that observed in the other composites.

**Figure 5:**
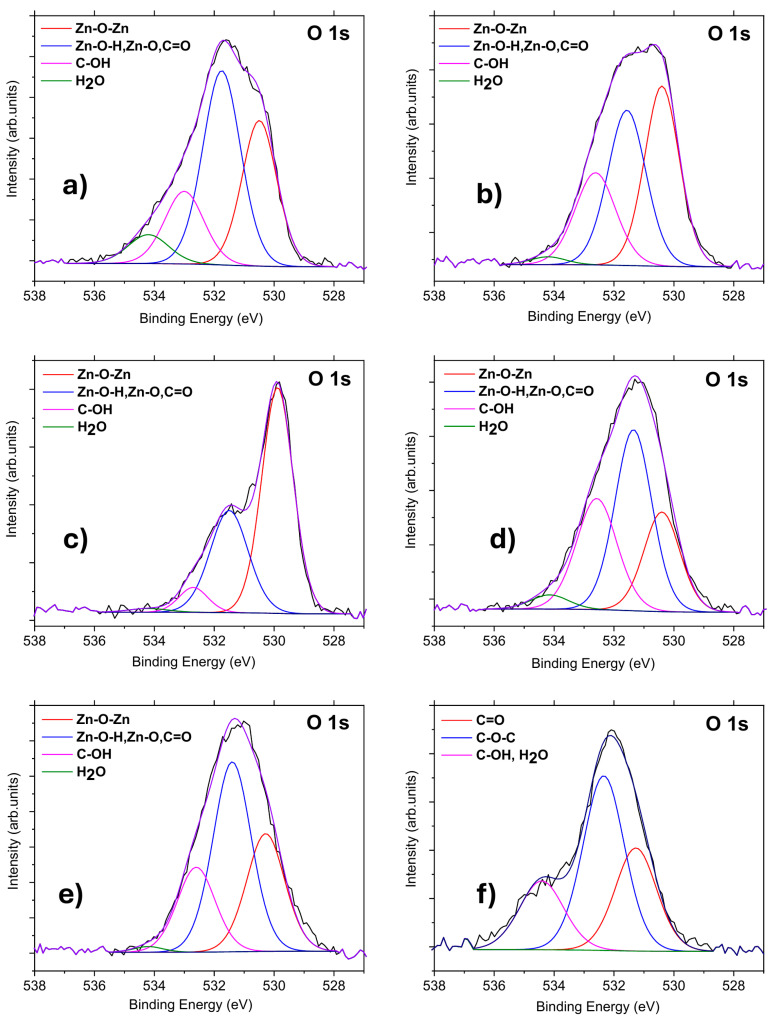
The O 1s XPS peaks of (**a**) ZnO reference sample, (**b**) ND-ZnO 5, (**c**) ND-ZnO 10, (**d**) ND-ZnO 15, (**e**) ND-ZnO 20, and (**f**) oxidized ND samples.

XPS analysis of O 1s peaks (Figure 5, Table 3) revealed that the O 1s peak of the ZnO reference sample appeared at 531.6 eV and shifted to a lower binding energy compared to the oxidized ND sample. The positions of the O 1s peaks in ND–ZnO 5, 15, and 20 were close to that of the ZnO reference, whereas the O 1s peak of ND–ZnO 10 was located at 530.0 eV, shifted by 1.6 eV relative to the reference. Moreover, the shape of the O 1s peak in ND–ZnO 10 differed from those of the other composites, which may have been attributed to uncompensated surface charge effects.

The O 1s peak of the ZnO reference was deconvoluted into three components [32]: lattice oxygen (Zn–O–Zn) at 530.5 ± 0.2 eV, nonlattice oxygen (Zn–OH, Zn–O, C=O) at 531.9 eV, and C–OH at 532.9 eV. An additional component at 534.4 ± 0.2 eV was assigned to adsorbed H_2_O. The oxidized ND sample was fitted with two main components [28,32], C=O (531.2 eV) and C–O (532.6 eV), together with C–OH and H_2_O contributions (534.4 ± 0.2 eV). The ND–ZnO composites (5, 10, 15, and 20) were deconvoluted into four components [28,32]: Zn–O–Zn (530.5 ± 0.2 eV), nonlattice oxygen (Zn–OH, Zn–O, C=O) at 531.9 eV, C–OH at 532.9 eV, and H_2_O at 534.4 ± 0.2 eV.

As summarized in Table 2 and Figure 4, the ND–ZnO composites exhibited higher proportions of Zn–O–Zn bonds compared to the ZnO reference (except ND–ZnO 15). In contrast, the H_2_O contribution (534.4 eV) decreased markedly in the composites, reaching as low as 2.1% compared to 6.9% in the ZnO reference. This reduction may have been related to differences in drying time before XPS measurement, which could influence water adsorption from the environment. No clear trend was observed for C=O and C–OH contributions across the composites relative to ZnO or oxidized ND samples. ND–ZnO 15 and ND–ZnO 20 exhibited similar O 1s peak shapes (FWHM = 2.6 eV) and low C–OH content (<5%). The O 1s peak of ND–ZnO 5 was the broadest (FWHM = 3.0 eV) and was located at 530.8 eV, suggesting a chemical composition close to that of the ZnO reference. Precise identification of C–O–C bonds in the composites remained challenging, as the fitted positions of C–OH (532.9 eV in ZnO reference) and C–O–C (532.6 eV in oxidized NDs) were very close. Overall, the O 1s peaks of the ND–ZnO composites exhibited similar positions and shapes to those of the ZnO reference, supporting comparable chemical environments.

**Figure 6:**
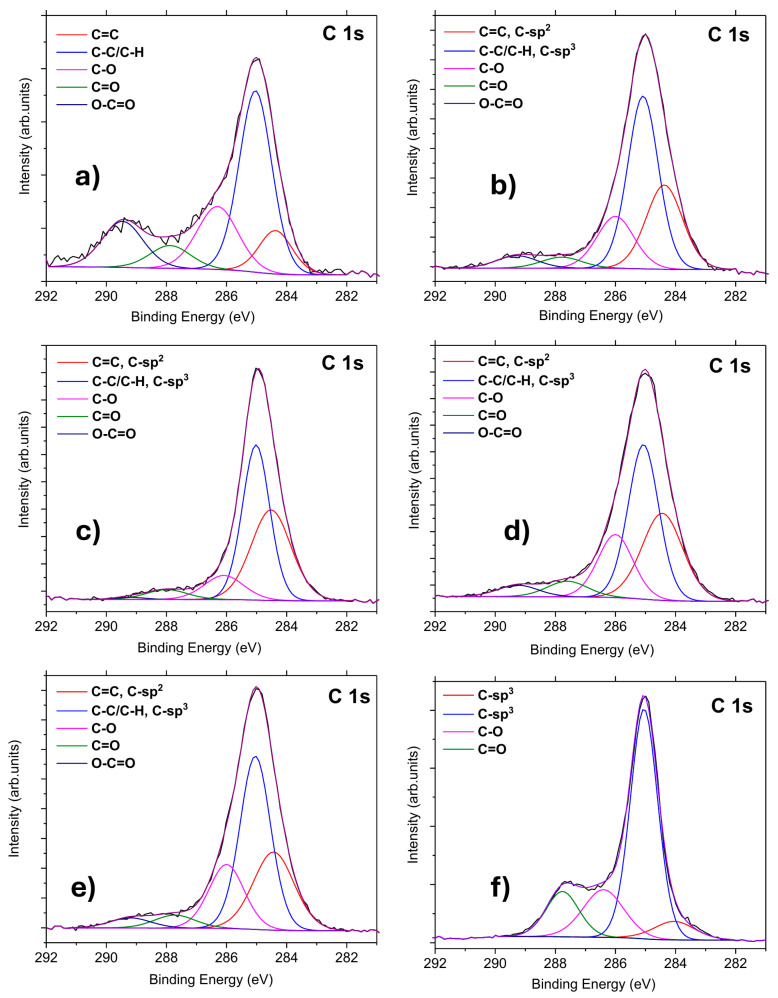
The C 1s XPS peaks of (**a**) ZnO reference, (**b**) ND-ZnO 5, (**c**) ND-ZnO 10, (**d**) ND-ZnO 15, (**e**) ND-ZnO 20, and (**f**) oxidized NDs. The measured data are marked as a black line.

C 1s spectra (Figure 6, Table 4) revealed that the ND–ZnO composites exhibited peak shapes comparable to those of the ZnO reference sample. The fitting of the C 1s peak in the ZnO reference was performed as follows [32]: 284.4 eV (C=C), 285.0 eV (C–C/C–H), 286.2 eV (C–O), 287.8 eV (C=O), and 289.4 eV (O–C=O). According to previous studies [30,40,41], the C 1s peak of oxidized ND samples was deconvoluted into four components: C–sp^2^ (284.2 eV), C–sp^3^ (285.3 eV), C–O (286.6 eV), and C=O (287.6 eV). In contrast, the ND–ZnO composites (5, 10, 15, and 20) were fitted with five components [30,32,40,41]: C=C (C–sp^2^, 284.4 eV), C–C/C–H (C–sp^3^, 285.0 eV), C–O (286.2 eV), C=O (287.8 eV), and O–C=O (289.4 eV).

As shown in Table 4 and Figure 6, the oxidized ND sample exhibited higher contributions from C–C/C–H (C–sp^3^) and C=C (C–sp^2^) bonds compared to the ZnO reference. The mixed ND–ZnO composites displayed increased amounts of C–C/C–H and C=C bonds relative to the reference sample. The concentration of C–C/C–H (C–sp^3^) bonds was comparable to that of the ZnO reference, whereas the proportion of C=C (C–sp^2^) bonds was significantly higher, up to 31% compared to less than 7% in the ZnO and oxidized ND samples. This enhancement was likely associated with graphitization induced by thermal treatment of the composites. Furthermore, the O–C=O bond content was below 5% in ND–ZnO samples, while the ZnO reference exhibited approximately 17%.

Among all ND–ZnO composites, the ND–ZnO 10 sample exhibits the most distinct XPS behavior, as reflected in Table 2, Table 3 and Table 4. In the Zn 2p region, ND–ZnO 10 shows a 0.5 eV shift in the Zn 2p_3_/_2_ peak toward lower binding energy, together with an unusually high proportion of Zn bulk bonds (51.6%) and a markedly reduced Zn–OH contribution (3.6%). This redistribution of Zn-related components suggests a modified Zn–O bonding environment and more efficient defect passivation at this composition.

The O 1s spectrum of ND–ZnO 10 also deviates from the other samples. It exhibits the highest fraction of lattice oxygen Zn–O–Zn (61.6%) and the lowest contributions from hydroxyl-related and carbon–oxygen species. The 1.6 eV shift in the O 1s peak toward lower binding energy indicates stronger Zn–O bonding and reduced surface defect density, consistent with improved electronic coupling at the ND–ZnO interface.

In the C 1s region, ND–ZnO 10 displays the highest proportion of sp^2^-hybridized carbon (31.5%), significantly exceeding both the ZnO reference and the other ND–ZnO composites. This enhancement points to the presence of graphitized surface domains on nanodiamonds that interact electronically with ZnO and act as efficient electron-accepting sites.

Taken together, these XPS features demonstrate that ND–ZnO 10 achieves an optimal balance between defect suppression, interfacial charge transfer, and chemical bonding. This unique combination of structural and electronic factors explains why ND–ZnO 10 exhibits the highest photocatalytic activity among all prepared composites.

### 3.4. XRD Characterization

**Figure 7:**
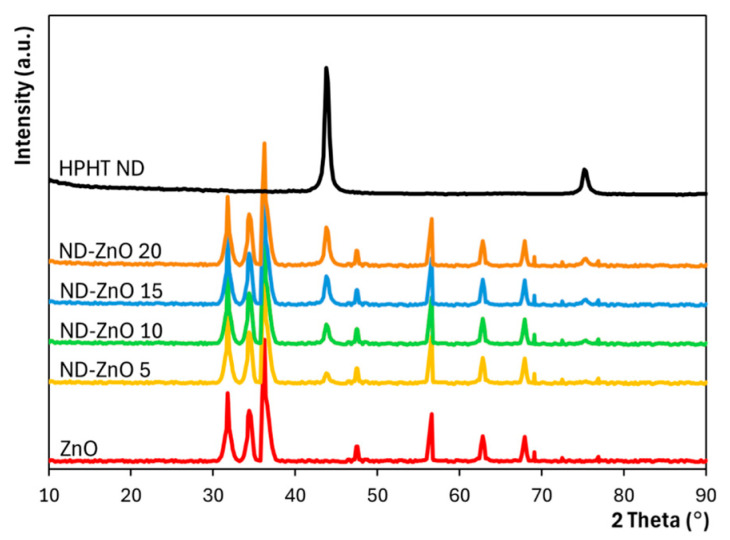
XRD patterns of synthesized ND–ZnO composites with different ND loadings, together with ZnO and oxidized HPHT ND.

X-ray diffraction (XRD) patterns of reference ZnO, oxidized HPHT nanodiamonds (NDs), and ND–ZnO composites with varying ND loadings (Figure 7) confirm the presence of both starting components in all synthesized composites. Each nanocomposite exhibits a strong characteristic trio of reflections corresponding to the wurtzite ZnO phase (space group P6_3_mc) at 2θ = 31.74° (100), 34.40° (002), and 36.22° (101) [43], along with additional ZnO-related peaks at 47.5° (102), 56.58° (110), 62.84° (103), and higher angles. Simultaneously, all ND–ZnO composites display the dominant diffraction peak of HPHT nanodiamond at 2θ = 43.8° (111) and a weaker signal at 75.6° (220), confirming the structural contribution of both ZnO and ND components.

### 3.5. Cathodoluminescence (CL) Spectroscopy

**Figure 8:**
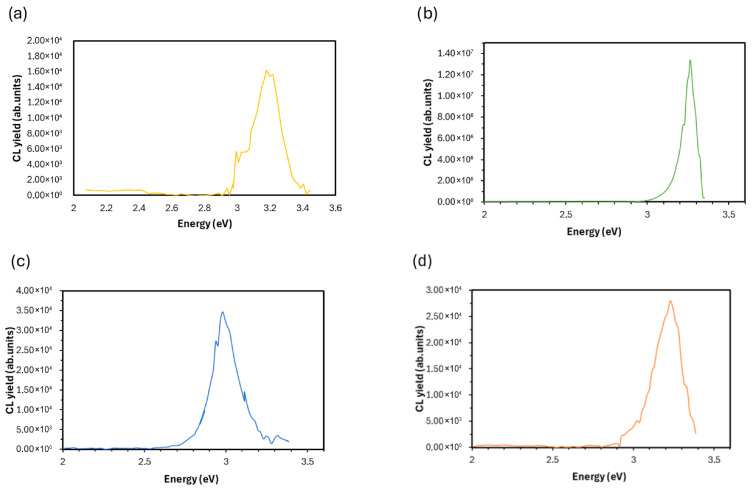
Cathodoluminescence (CL) spectra of (**a**) ND–ZnO 5, (**b**) ND–ZnO 10, (**c**) ND–ZnO 15, and (**d**) ND–ZnO 20 composites.

The cathodoluminescence (CL) spectra of the ND–ZnO composites (Figure 8) revealed dominant near-band-edge (NBE) emissions in the UV region across all samples. The NBE maximum appeared at ~3.2–3.3 eV, consistent with the direct band gap of wurtzite ZnO at room temperature [44,45]. Minor variations in the NBE peak position among the samples can be attributed to differences in defect density, local strain, and band-tail states induced by structural disorder or surface effects [46]. In addition to the NBE emissions, a broad visible luminescence band extending from ~2.0 to 3.0 eV was observed, commonly associated with intrinsic defects such as oxygen vacancies, zinc interstitials, and related surface states [45,47]. These defect-related transitions compete with near-band-edge recombination and may contribute to slight broadening or subtle shifts in the apparent optical gap derived from CL measurements [48].

Within this visible region, the main defect-related feature was a blue band centered around ~3.0 eV, typically attributed to Zn interstitials (Zn*i*) [49], whereas O-related defects expected near 2.15 eV were weak and did not significantly contribute to the emissions. Clear differences were observed among the samples in the relative intensity of excitonic emissions. ND–ZnO 5 and ND–ZnO 10 exhibited spectra dominated by sharp excitonic peaks, particularly pronounced in ND–ZnO 10, indicating high crystallinity and reduced non-radiative defect density. Conversely, ND–ZnO 15 and ND–ZnO 20 showed mixed contributions from excitonic and Zn*i*-related defect bands, reflecting lower crystalline quality and higher defect density.

### 3.6. Photocatalytic Activity

The photocatalytic activity of ND–ZnO composites was evaluated through the degradation of methylene blue (MB) under UV irradiation. The efficiency was assessed by monitoring the variation in *ln(c_0_/c)* as a function of irradiation time. The degradation kinetics followed a pseudo-first-order reaction, described by the Langmuir–Hinshelwood model [50,51], consistent with previous reports [52]. The kinetic behavior is expressed by Equation (1):

(1) $$\ln\frac{c_{0}}{c}=k_{s}\cdot t$$

where c_0_ and *c* denote the initial and instantaneous MB concentrations, respectively; *k_s_* is the rate constant; and *t* is the irradiation time.

**Figure 9:**
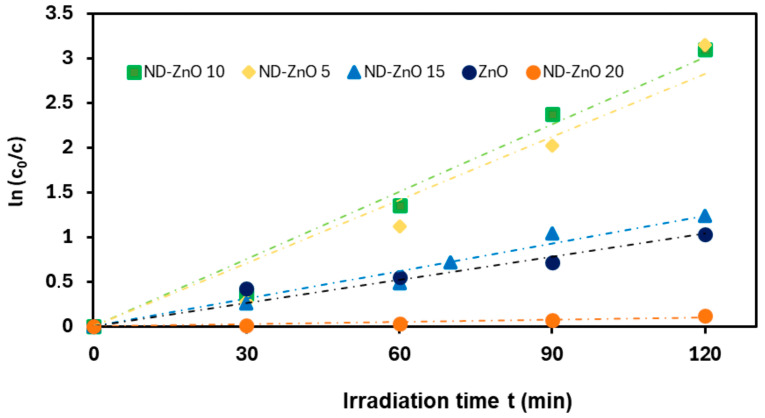
Time dependency of the photocatalytic MB degradation. The straight lines represent the linear fit from the Langmuir–Hinshelwood model.

Linear fits (Figure 9) and the corresponding rate constants (Table 5) revealed that ND–ZnO 10 exhibited the highest photocatalytic activity, with a rate constant approximately three times higher than pristine ZnO. ND–ZnO 5 also showed enhanced activity, whereas ND–ZnO 15 and ND–ZnO 20 displayed significantly lower efficiencies, comparable or inferior to pristine ZnO.

#### Reusability Test

**Figure 10:**
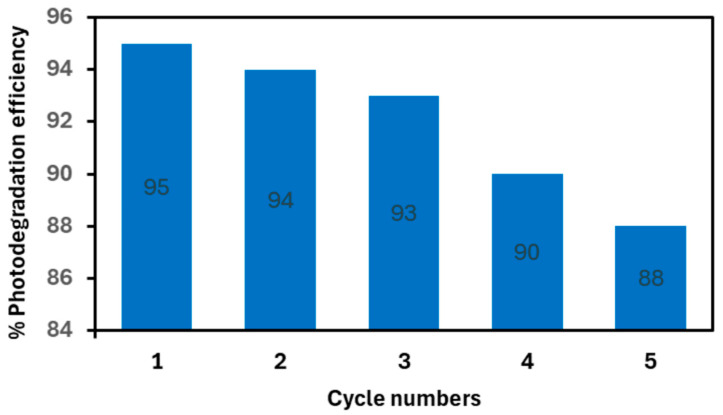
Reusability of the ND–ZnO 10 composite for the photocatalytic degradation of MB under UV irradiation over five consecutive cycles.

The reusability of the most efficient composite (ND–ZnO 10) was evaluated in five consecutive photocatalytic cycles of MB degradation under identical experimental conditions. As shown in Figure 10, the photocatalytic efficiency decreased slightly from 95% in the first cycle to 88% after the fifth cycle. This minor reduction demonstrates that ND–ZnO 10 retained the majority of its photocatalytic activity over repeated use.

## 4. Discussion

The incorporation of oxidized high-pressure high-temperature (HPHT) nanodiamonds into ZnO nanostructures was shown to significantly modulate their structural, optical, and photocatalytic properties. The enhanced photocatalytic activity observed for ND–ZnO 5 and ND–ZnO 10 can be explained by two complementary mechanisms: (i) defect passivation, evidenced by suppressed defect-related luminescence in cathodoluminescence spectra, and (ii) efficient interfacial charge transfer facilitated by sp^2^ carbon domains in nanodiamonds acting as electron acceptors. Similar defect passivation effects have been reported for ZnO–graphene composites, where graphene layers reduce non-radiative recombination and extend carrier lifetimes [53]. These effects reduce the density of recombination centers, prolong carrier lifetimes, and enable more efficient generation of reactive oxygen species, thereby improving photocatalytic degradation efficiency. A broader comparison of ZnO–carbon hybrid photocatalysts reported for MB degradation is provided in Table S1 (Supplementary Materials). The XRD analysis demonstrates that dehydration condensation successfully produced ND-ZnO composites in which both crystalline phases are preserved. The ZnO reflections remain well defined, indicating that the wurtzite structure is maintained across all compositions.

The enhanced photocatalytic activity of ND–ZnO composites can also be interpreted in terms of reactive oxygen species (ROS) generation. Although direct ESR or fluorescence-based trapping measurements were not performed, several indirect but mechanistically consistent indicators supported the involvement of superoxide (O_2_•^−^) and hydroxyl (•OH) radicals. Cathodoluminescence spectra of ND-modified samples revealed a pronounced suppression of deep-level defect emissions, indicating reduced electron–hole recombination and increased availability of charge carriers for interfacial redox reactions. Oxygen vacancies and surface hydroxyl groups—key sites responsible for the formation of O_2_•^−^ and •OH radicals in ZnO-based photocatalysts—are known to be strongly influenced by carbonaceous modifiers [54,55]. Furthermore, previous studies have shown that HPHT and detonation nanodiamonds containing sp^2^-hybridized surface domains act as efficient electron acceptors, facilitating electron transfer to dissolved oxygen and promoting superoxide formation [56,57]. These literature-supported mechanisms, together with the observed enhancement in photocatalytic performance, provide strong indirect evidence that ND incorporation promotes ROS generation in ND–ZnO composites.

The dependence of photocatalytic performance on ND loading highlights the importance of optimizing composite composition. While moderate incorporation of nanodiamonds improves activity, higher ND contents (ND–ZnO 15 and ND–ZnO 20) lead to a pronounced decrease in efficiency. This decline can be attributed to excessive carbon concentration, which promotes aggregation of nanodiamond domains and partial shielding of ZnO active sites, thereby reducing the accessible surface area and limiting UV light absorption. XPS analysis (Table 2, Table 3 and Table 4) further supports this interpretation: ND–ZnO 20 exhibits a lower fraction of lattice oxygen, higher contributions from defect-related oxygen species, and a reduced sp^2^ carbon signal compared to ND–ZnO 10. These structural and electronic features indicate inefficient interfacial charge transfer and enhanced electron–hole recombination. Such behavior is consistent with previous reports on ZnO/carbon nanotube systems, where excessive carbon loading caused aggregation and diminished photocatalytic activity [58]. Together, these effects explain the extremely low photocatalytic performance of the ND–ZnO 20 composite.

Such findings emphasize that optimal ND:ZnO ratios are required to balance defect suppression and interfacial charge transfer. The elemental ratios obtained from EDX do not directly reflect the nominal ND:ZnO weight ratios because EDX only probes the near-surface region, where nanodiamond aggregates preferentially accumulate. This leads to an apparent enrichment of carbon relative to zinc, consistent with the surface-sensitive XPS results showing increased C-related components and modified Zn–O bonding. The ND–ZnO 10 composite, which exhibits the most balanced surface composition in EDX and the strongest lattice oxygen contribution in XPS, also shows the lowest defect-related luminescence in CL. These correlated observations explain why ND–ZnO 10 achieves the highest photocatalytic activity, while higher ND loadings introduce carbon-rich domains that increase recombination and reduce efficiency.

All photocatalytic experiments in this work were carried out under fixed and standardized conditions, with identical catalyst loading and initial MB concentration. Total organic carbon (TOC) analysis was not included in the present study, as the aim was to compare the relative photocatalytic activities of the ND–ZnO composites under identical conditions; however, TOC represents an important direction for future work focused on evaluating complete mineralization of organic pollutants. These parameters were intentionally kept constant to ensure that the observed differences in degradation efficiency reflected intrinsic material properties rather than variations in reaction environment. Since catalyst dosage and dye concentration are known to influence light absorption, availability of active sites, and mass transfer processes, maintaining uniform reaction conditions therefore allows for a direct assessment of how HPHT nanodiamond incorporation modulates the structural and surface chemical features of ZnO that govern photocatalytic performance.

The reusability tests confirmed the practical potential of ND–ZnO 10, which retained most of its activity after five consecutive cycles. The slight decrease in efficiency can be attributed to partial catalyst loss or aggregation during recovery, but overall stability remained high. Comparable stability has been documented for ZnO–graphene oxide composites, which maintained high photocatalytic activity over multiple cycles of dye degradation [59]. The stability of the ND–ZnO composites during repeated photocatalytic cycles also indicated that metal leaching was negligible under the applied reaction conditions. ZnO is known to remain structurally stable in neutral aqueous media, with only minimal Zn^2+^ release reported under such conditions [60,61], and the incorporation of HPHT nanodiamonds did not introduce additional metal species that could undergo dissolution. The preserved photocatalytic activity of ND–ZnO 10 after multiple cycles supported the conclusion that Zn^2+^ release into solution was minimal. This behavior is consistent with previous reports on ZnO-based hybrid photocatalysts, where stable performance over repeated cycles correlates with negligible leaching and robust interfacial bonding [54,55]. This robustness underscores the suitability of HPHT ND–ZnO composites for repeated use in environmental remediation.

At the level of heterojunction interactions, ND–ZnO systems exhibit mechanisms similar to those reported for graphene–ZnO [62] and fullerene–ZnO [63] composites, where the carbon component provides efficient pathways for electron transport and suppresses electron–hole recombination [13,53,58]. Fullerene–ZnO hybrids, for example, have demonstrated efficient charge separation due to π-conjugated domains acting as electron sinks [58]. These literature examples support the assertion that HPHT nanodiamonds, particularly those containing mildly graphitized regions, can act as effective electron sinks, thereby contributing to efficient charge separation and improved photocatalytic performance. The choice of HPHT nanodiamonds in this study is motivated by their well-defined crystalline structure, low defect density, and stable surface chemistry, which together promote efficient charge transfer and suppress electron–hole recombination in ZnO-based photocatalysts. In contrast, detonation nanodiamonds (DNDs) typically contain a high concentration of structural defects, graphitic shells, and heterogeneous oxygen-containing groups, which may act as recombination centers and reduce photocatalytic efficiency. Although DND–ZnO composites have been reported for the degradation of phenol, toluene, and methyl orange, no studies have evaluated their performance toward MB degradation, preventing a direct photocatalytic comparison. These material differences highlight the advantages of HPHT NDs as more controlled and stable carbon modifiers for ZnO.

Taken together, the results provide new insights into defect engineering strategies for ZnO-based photocatalysts. Unlike detonation nanodiamonds, which suffer from unstable surface chemistry and high defect density, HPHT nanodiamonds offer a more controlled and tunable platform for enhancing photocatalytic activity. The demonstrated synergy between defect suppression and charge transfer establishes HPHT ND–ZnO composites as promising candidates for sustainable wastewater treatment and related environmental applications. These findings align with broader trends in carbon–ZnO hybrid research, where tailored carbon nanostructures (graphene, CNTs, fullerenes) have consistently improved photocatalytic efficiency through defect modulation and charge transfer [13,53,58,59].

**Figure 11:**
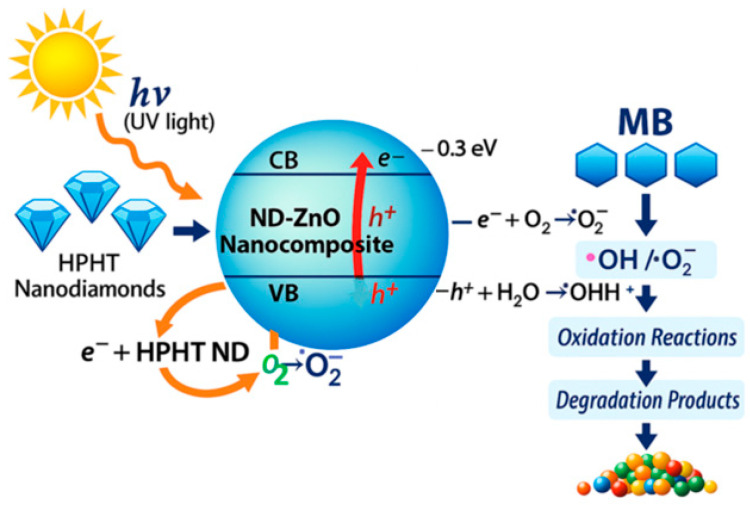
Schematic illustration of the photocatalytic degradation mechanism of methylene blue (MB) using the HPHT nanodiamond-modified ZnO nanocomposite under UV irradiation. The arrows indicate the migration of photogenerated charge carriers (e^−^/h⁺) and the formation of reactive oxygen species involved in MB degradation.

To provide a clearer interpretation of the enhanced photocatalytic performance of the ND–ZnO composites, a schematic illustration of the methylene blue (MB) degradation mechanism under UV irradiation has been included (Figure 11). Upon exposure to UV light (λ = 365 nm), ZnO absorbs photons and generates electron–hole pairs according to:

ND–ZnO + hν_UV_ → e_CB_^−^+ h_VB_^+^

The incorporation of HPHT nanodiamonds facilitates charge separation as they act as efficient electron acceptors, thereby suppressing electron–hole recombination at the ZnO surface. The photogenerated electrons subsequently react with dissolved oxygen to form superoxide radicals:

e^−^ + O_2_ → •O_2_^−^

while the photogenerated holes oxidize water molecules to produce hydroxyl radicals:

h^+^ + H_2_O → •OH + •H^+^

Both reactive oxygen species (•O_2_^−^ and •OH) participate in the oxidative degradation of MB:

MB + (•OH, •O_2_^−^) → degradation products.

This mechanism is consistent with the observed enhancement in photocatalytic activity, particularly for the ND–ZnO 10 composite, where the optimized nanodiamond content promotes efficient interfacial charge transfer and maximizes the generation of reactive oxygen species. The synergistic interaction between ZnO and HPHT nanodiamonds therefore plays a central role in accelerating MB degradation under UV irradiation.

A comprehensive comparison of ZnO-based photocatalysts modified with various carbon nanomaterials is provided in Table S1 (Supplementary Materials), highlighting the advantages of the HPHT nanodiamond-modified ZnO system developed in this work.

## 5. Conclusions

ND–ZnO composites with varying nanodiamond loadings were successfully synthesized and thoroughly characterized. Moderate incorporation of HPHT nanodiamonds (ND:ZnO weight ratios of 0.05–0.10) effectively suppressed defect-related luminescence and enhanced near-band-edge emissions, indicating defect passivation and improved interfacial charge transfer. These changes directly influenced the photocatalytic performance, with ND–ZnO 10 showing the highest activity in methylene blue degradation, with a rate constant nearly three times that of pristine ZnO. Conversely, higher ND loadings (0.15–0.20) increased defect density and decreased photocatalytic efficiency, highlighting the need to optimize ND content. Reusability tests confirmed that ND–ZnO 10 maintained most of its photocatalytic activity after five consecutive cycles, demonstrating its stability and practical potential.

The novelty of this work lies in using HPHT nanodiamonds, which offer a more controlled and adjustable platform compared to conventional detonation nanodiamonds, and in the innovative application of cathodoluminescence spectroscopy to link defect passivation with photocatalytic efficiency.

These results underscore the potential of HPHT ND–ZnO composites as efficient, reusable photocatalysts and provide new insights into defect engineering strategies and practical solutions for environmental cleanup.

## Figures and Tables

**Table 1:** The atomic concentration of chemical elements in the samples calculated from HR XPS spectra.

Sample Name	Atomic Concentration of Chemical Elements (at.%)				
	O	C	Zn	N	Si
ZnO	45.2	32.8	19.5	2.5	-
ND	10.2	89.2	-	-	0.6
ND-ZnO 5	34.4	52.3	13.3	-	-
ND-ZnO 10	27.6	55.9	16.5	-	-
ND-ZnO 15	30.1	57.1	12.8	-	-
ND-ZnO 20	26.0	67.2	6.8	-	-

**Table 2:** Binding energies and relative peak-area contributions (%) of fitted Zn 2p components.

Sample Name	Position [eV]	Relative Peak Area (%)		
		Zn Bulk	Zn–O	Zn–OH
ZnO	1021.6	2.4	80.7	16.9
ND-ZnO 5	1021.6	2.2	89.2	8.6
ND-ZnO 10	1021.1	51.6	44.8	3.6
ND-ZnO 15	1021.8	6.1	84.1	9.8
ND-ZnO 20	1021.8	10.1	71.7	18.2

**Table 3:** Binding energies and relative peak-area contributions (%) of fitted O 1s components.

Sample Name	Position [eV]	Relative Peak Area (%)			
		Zn–O–Zn	Zn–O–H, Zn–O, (C=O)	C–OH, (C–O–C)	H_2_O, (C–OH)
ZnO	531.6	31.3	43.2	18.6	6.9
ND	532.0	-	23.0	56.6	20.4
ND-ZnO 5	530.8	38.9	51.6	7.1	2.4
ND-ZnO 10	530.0	61.6	34.1	1.7	2.6
ND-ZnO 15	531.3	24.2	60.7	10.7	4.4
ND-ZnO 20	531.1	32.9	56.4	8.6	2.1

**Table 4:** Relative peak-area contributions (%) of fitted C 1s components (calibrated to 285.0 eV).

Sample Name	Relative Peak Area (%)				
	C=C, C–sp^2^	C–H/C–C, C–sp^3^	C–O	C=O	O–C=O
ZnO	4.2	53.1	17.4	8.5	16.8
ND	6.6	60.7	16.9	15.8	-
ND-ZnO 5	28.0	50.2	12.1	4.7	5.0
ND-ZnO 10	31.5	54.7	7.5	5.3	1.0
ND-ZnO 15	25.8	48.6	15.9	5.2	4.5
ND-ZnO 20	21.9	53.2	15.6	5.2	4.1

**Table 5:** Comparison of rate constants k and correlation coefficients R^2^ for a series of ND-ZnO and ZnO photocatalysts.

Photocatalyst	*k* (min^−1^)	R^2^
ND-ZnO 5	0.0235	0.9751
ND-ZnO 10	0.0251	0.9886
ND-ZnO 15	0.0103	0.9903
ND-ZnO 20	0.0009	0.9428
ZnO	0.0087	0.9846

## Data Availability

The original data presented in the study are openly available in [Zenodo] at [https://doi.org/10.5281/zenodo.18478460], accessed on 23 January 2026.

## Supplementary Materials

The following supporting information can be downloaded at https://www.mdpi.com/article/10.3390/ma19030609/s1: Figure S1. Comparison of the normalized A XPS survey spectra and B magnification regions (390-410 eV) of the survey spectra of reference ZnO and ND-ZnO 10 samples. Figure S2. The high-resolution N 1s peak detected on the surface of the reference ZnO sample. Table S1. ZnO based photocatalysts modified with various carbon nanomaterials for the photocatalytic degradation of methylene blue (MB) under UV irradiation. References [64,65,66,67,68,69,70,71] are cited in the Supplementary Materials.
