# Supplementary material for: High-Pressure High-Temperature Nanodiamond-Modified ZnO Nanocomposites as Promising Photocatalysts: Synthesis and Characterization
Source: Materials (Basel). 2026 Feb 4;19(3):609. doi: 10.3390/ma19030609 (PMC12899666; doi:10.3390/ma19030609)
Supplement: Supplementary file 1 [file materials-19-00609-s001.zip › materials-4067982-supplementary.pdf]

High-Pressure High-Temperature nanodiamond-modified ZnO nanocomposites as promising photocatalysts: synthesis and characterization

Julia Micova [1,*], Natalia Kosutova [1], Miroslav Cavojsky [2], Anna Artemenko [3], Zdenek Remes [3,*], Bruno Masenelli [4], Gilles Ledoux [5]

1 Institute of Chemistry Slovak Academy of Sciences, Dúbravská cesta 5807/9, 845 38 Bratislava, Slovakia

2 Institute of Materials and Machine Mechanics Slovak Academy of Sciences, Dúbravská cesta 9/6319, 845 38,

3 Institute of Physics of the Czech Academy of Sciences, Na Slovance 1999/2, 182 00 Prague, Czech Republic

4 INSA Lyon, Ecole Centrale de Lyon, CNRS, Universite Claude Bernard Lyon 1, CPE Lyon, INL, UMR5270, 69621 Villeurbanne, France

5 Universite Claude Bernard Lyon 1, CNRS, Institut Lumière Matière, UMR5306, F-69100, Villeurbanne, France

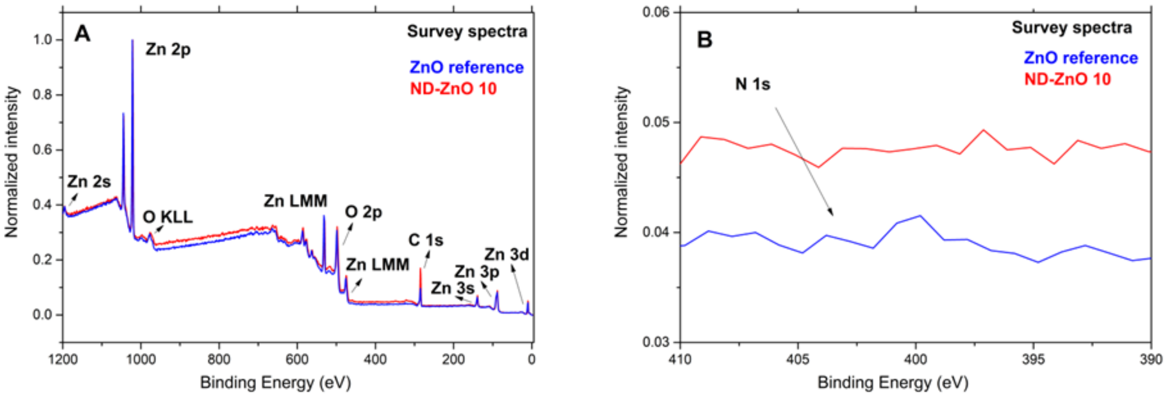

Figure S1. Comparison of the normalized A XPS survey spectra and B magnification regions (390-410 eV) of the survey spectra of reference ZnO and ND-ZnO 10 samples.

The comparison of the normalized XPS survey spectra of reference ZnO and ND-ZnO 10 samples demonstrated in Figure S1 revealed the low signal of nitrogen on the surface of the reference ZnO (see Figure S1B, magnification region). In addition, the high-resolution N 1s peak was measured in the case of reference ZnO, as shown in Figure S2, and XPS analysis revealed the presence of ~ 2.5 at.% (Table 1) of nitrogen on the surface of the reference ZnO. The intensity of the N 1s peak was rather low and the shape was noisy; however, it could be assumed that the peak was located at about 399.9 eV and could be a reference to some amide groups. Thus, the presence of a low amount of nitrogen in reference ZnO could be attributed to the adsorption of nitrogen-containing species from the atmosphere during the sample handling. As for the ND-ZnO 10 sample, no significant evidence of N 1s peak signal was observed in the XPS survey spectra (probably due to the rather low amount of N and the detection limits of the used XPS spectrometer). This confirmed that negligible nitrogen-containing species were introduced during the preparation of the ND–ZnO composites and that nitrogen was not incorporated into the ZnO lattice.

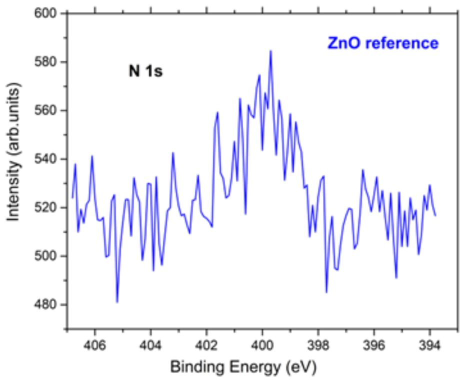

Figure S2. The high-resolution N 1s peak detected on the surface of the reference ZnO sample.

Table S1. ZnO-based photocatalysts modified with various carbon nanomaterials for the photocatalytic degradation of methylene blue (MB) under UV irradiation.

Photocatalyst system	Carbon modifier	MB degradation efficiency	Reference
C-doped ZnO	Carbon from plant extract	~62%	[64]
ZnO/CDots	Carbon dots	~80%	[65]
ZnO/ACF	Activated carbon fibers	~78–99%	[66]
ZnO/CNT	Carbon nanotubes	~90–99%	[67]
ZnO/MWCNT	Multi-walled CNT	~99%	[68]
ZnO–rGO	Reduced graphene oxide	~86–99%	[69,70]
ZnO–GO	Graphene oxide	~75–99%	[71]
**HPHT ND–ZnO (this work)**	HPHT nanodiamond	**~98%**	This work

References

64 Huong, L.M.; Cong, C.Q.; Dat, N.M.; Hai, N.D.; Nam, N.T.H.; An, H.; Tai, L.T.; Do Dat, T.; Dat, N.T.; Phong, M.T.; et al. Green Synthesis of Carbon-Doped Zinc Oxide Using Garcinia Mangostana Peel Extract: Characterization, Photocatalytic Degradation, and Hydrogen Peroxide Production. *Journal of Cleaner Production* **2023**, *392*, 136269, doi:10.1016/j.jclepro.2023.136269.

65. Maddu, A.; Meliafatmah, R.; Rustami, E. Enhancing Photocatalytic Degradationof Methylene Blue Using ZnO/Carbon DotsNanocomposite Derived From Coffee Grounds. *Pol. J. Environ. Stud.* **2020**, *30*, 273–282, doi:10.15244/pjoes/120156.

66. Albiss, B.; Abu-Dalo, M. Photocatalytic Degradation of Methylene Blue Using Zinc Oxide Nanorods Grown on Activated Carbon Fibers. *Sustainability* **2021**, *13*, 4729, doi:10.3390/su13094729.

67. Mostafa, A.M.; Mwafy, E.A.; Toghan, A. ZnO Nanoparticles Decorated Carbon Nanotubes via Pulsed Laser Ablation Method for Degradation of Methylene Blue Dyes. *Colloids and Surfaces A: Physicochemical and Engineering Aspects* **2021**, *627*, 127204, doi:10.1016/j.colsurfa.2021.127204.

68. Liu, P.; Guo, Y.; Xu, Q.; Wang, F.; Li, Y.; Shao, K. Enhanced Photocatalytic Performance of ZnO/Multi-Walled Carbon Nanotube Nanocomposites for Dye Degradation. *Ceramics International* **2014**, *40*, 5629–5633, doi:10.1016/j.ceramint.2013.10.157.

69. Xue, B.; Zou, Y. High Photocatalytic Activity of ZnO–Graphene Composite. *Journal of Colloid and Interface Science* **2018**, *529*, 306–313, doi:10.1016/j.jcis.2018.04.040.

70 Marcano, D.C.; Kosynkin, D.V.; Berlin, J.M.; Sinitskii, A.; Sun, Z.; Slesarev, A.; Alemany, L.B.; Lu, W.; Tour, J.M. Improved Synthesis of Graphene Oxide. *ACS Nano* **2010**, *4*, 4806–4814, doi:10.1021/nn1006368.

71. Rokhsat, E.; Akhavan, O. Improving the Photocatalytic Activity of Graphene Oxide/ZnO Nanorod Films by UV Irradiation. *Applied Surface Science* **2016**, *371*, 590–595, doi:10.1016/j.apsusc.2016.02.222.
